# De-anthropomorphizing the mind: life as a cognitive spectrum in a unified framework for biological minds

**DOI:** 10.3389/fnsys.2026.1730097

**Published:** 2026-01-22

**Authors:** Gordana Dodig-Crnkovic

**Affiliations:** 1 Division of Computer Science and Software Engineering, School of Innovation, Design and Engineering, Mälardalen University, Västerås, Sweden; 2 Department of Computer Science and Engineering, Chalmers University of Technology, Gothenburg, Sweden

**Keywords:** awareness, cognition, consciousness, intelligence, mind, sentience

## Abstract

Cognition, sentience, intelligence, awareness, and mind are often treated as distinct phenomena that emerge only at higher levels of biological organization, typically associated with nervous systems or human cognition. However, empirical research increasingly demonstrates learning, memory, adaptive behavior, and goal-directed regulation across a wide range of living systems, including single cells, tissues, and organisms without brains. This paper proposes a unifying framework in which cognition is understood as an organizational property of living systems, grounded in information embodied in their physical structures and in their ongoing interactions with the environment. Within this info-computational (ICON) perspective, living systems engage in behavior, learning, and anticipation by dynamically transforming embodied information through distributed, physically realized processes that support viability and self-maintenance. These processes are present from the onset of life and become progressively more integrated and temporally extended with increasing biological organization. The framework provides explanatory continuity across biological scales and clarifies how complex forms of cognition, awareness, and mind arise as elaborations of basic life-regulatory dynamics. It generates empirically grounded, testable implications for basal cognition, developmental biology, and embodied artificial systems, in the domains such as morphogenetic regulation, bioelectric control, and embodied physical architectures where its implications can be tested.

## Introduction

The recent impressive advancements in AI once again bring to the forefront the question of our understanding of intelligence in humans, animals, and artificial systems. It is evident that our current definitions of fundamental terms such as cognition, sentience, intelligence, awareness/consciousness, and mind are insufficient, often leading to confusion and conceptual muddles.

The debate continues: some claim that ChatGPT is already conscious, while others argue that this is impossible, given its lack of the fundamental cognitive architecture that enables human consciousness. Yet, ChatGPT can engage in conversation with humans in an impressively convincing way. If the Turing test were applied at this stage, it would likely pass as intelligent. But is it conscious? Are animals conscious? What about bacteria?

Apart from a few panpsychists who believe that consciousness permeates the universe, the rest of us struggle with vague and inconsistently defined notions of intelligence.

The aim of this paper is to explore the concepts of cognition, sentience, intelligence, awareness/consciousness, and mind, and to develop a unified theoretical framework applicable to all living systems. Previous work has motivated such unification on empirical grounds, particularly through observations of cognitive-like behavior across biological scales (Dennett, 2017; Levin, 2022, 2023). However, these insights have not yet been integrated into a systematic, explanatory framework that clarifies how these capacities relate to one another within a single coherent account.

The present work proposes an info-computational framework (ICON) to address this gap, offering a coherent account of how cognition and related phenomena arise as organizational properties of living systems. Beyond its explanatory value for biological cognition, this unification has implications for technology: since nature has historically served as a source of inspiration for engineering and design, clearer conceptual foundations may inform the development of artificial intelligence, bio-inspired systems, and other cognitive technologies.

Cognition, intelligence, and consciousness exist on a continuum, with different life forms displaying distinct abilities shaped by their biology, evolution, and environment (Lyon, 2015; Adamatzky, 2022; Chis-Ciure and Levin, 2025; Ginsburg and Jablonka, 2019; Jablonka et al., 2014; Carrasco-Pujante et al., 2021; Boussard et al., 2021). Different kinds of living organisms possess different forms of cognition, sentience, intelligence, and awareness/consciousness. Instead of thinking in binary terms (conscious vs. non-conscious, intelligent vs. non-intelligent), we can explore how various organisms display different cognitive traits suited to their ecological roles. According to Maturana and Varela (1980), all living systems are cognitive by virtue of their self-maintaining, adaptive organization in ongoing structural coupling with the environment. Cognition in this naturalized sense does not presuppose nervous systems.

The fundamental question we can ask is: In a naturalist framework, how do mind and intelligence emerge in nature, and how do they relate to artificial cognition and intelligence?

## Bridging material and mental from biological to artifactual

### Proto-cognition and biological cognition

The traditional divide between inert matter and conscious mind is dissolving across disciplines. In fields as diverse as soft robotics, neurophenomenology, and bioengineering, researchers increasingly observe cognitive-like behaviors in non-neural systems. The discovery of sensing materials and intelligent matter demonstrates that the building blocks of life are not merely reactive but capable of functional evaluation, memory, and adaptive reorganization (Kaspar et al., 2021; Wang et al., 2020; Dixon et al., 2021; Hu et al., 2019).

This paper situates these findings within a continuum of cognition, arguing that the properties and behaviors of simple physical systems precede the basal cognition observed in living cells (Lyon et al., 2021; Jin et al., 2024) and, ultimately, the complex subconscious and conscious operations within the human body. In doing so, we offer a naturalistic, embodied, biologically inspired framework that may be used to inform the design of adaptive, embodied systems relevant to Artificial Intelligence (AI).

From its inception, AI was envisioned as an artifactual counterpart to human intelligence with the prospect of eventually surpassing it. The development of AI and research in human cognition have progressed concurrently and interactively. As Kari and Rozenberg (2008) explain, the learning process is two-way: while we design new technological systems, these systems, in turn, provide tools to test and refine our understanding of human mind and intelligence.

### From sensing materials with memory to proto-cognition

Materials like shape-memory alloys, piezoelectric polymers, and neuromorphic gels are capable of storing and processing information along with environmental interactions (Chortos and Bao, 2014; Prakash et al., 2023). These materials exhibit behaviors that, while non-conscious, are valence-sensitive as they respond differently to different stimuli in ways that resemble biological preference or avoidance.

Recent developments in neuromorphic engineering suggest that intelligent matter (Kaspar et al., 2021) can exhibit learning-like and adaptive responses, suggesting the idea that *material substrates can support basic cognitive architectures*. These materials provide a physical and conceptual basis for proto-cognition, i.e., early forms of information integration and action selection that precede life and scaffold its emergence.

Antecedent proto-cognitive processes in non-living matter do not imply cognition to such systems. Proto-cognition denotes physical and chemical precursor dynamics, such as adaptive material responses and constraint-driven reconfiguration, that enables the self-organization of living organisms but lack self-maintenance and intrinsic normativity. Cognition proper, as defined here, is restricted to living systems.

### Somatic cell cognition: distributed intelligence in the body

New research on cellular level shows memory as general property of cells. Cellular memory in non-neural cells involves corresponding molecular machinery previously found in neurons. Work of Kukushkin et al. (2024) experimentally demonstrates memory-like gene activation effects in non-neural cells, showing conserved molecular mechanisms underlying cellular learning. At the epigenetic level, cellular memory is supported by the propagation of DNA methylation and histone modifications through cell division, maintaining transcriptional programs essential for cell identity and differentiation (Espinosa-Martínez et al., 2024). This epigenetic cycle allows cells to “remember” environmental signals long after they cease, enabling stable phenotypic states and plastic responses. Further, microtubules and microtubule-associated proteins in cells contribute to intracellular signaling and structural plasticity relevant to memory consolidation and information storage (Flores and Liester, 2024). Disruption of these components can impair memory-related functions.

These findings characterize cellular memory as a multilayered phenomenon involving gene regulation, signaling networks, and epigenetic mechanisms, extending the concept of memory beyond the nervous system to all cell types. This has implications for understanding development, disease, and systemic embodied cognition.

Individual somatic cells in multicellular organisms demonstrate capacities for decision-making and learning at the biochemical and biophysical level. Immune cells, for example, evaluate threat, adapt responses based on past encounters, and communicate dynamically with other systems (Prinz and Priller, 2017). Stem cells integrate environmental cues to determine differentiation pathways, a process requiring multi-factorial assessment and response (Whited and Levin, 2019).

Michael Levin’s work on bioelectric signaling and morphogenetic fields reveals that tissues and organs participate in distributed information processing (computation), enabling body-wide decision-making during regeneration and development. These examples of somatic cognition are operating outside conscious awareness but are essential to adaptive behavior.

### Whole-body cognition and the subconscious as embodied mind

As multicellular coordination scales up, it gives rise to body-level intelligence that shapes affective states, intuitive actions, and physiological regulation. Neuroscientific models of interoception (Barrett and Simmons, 2015; Damasio, 1999) and enactivism (Varela et al., 1991) emphasize that conscious experience arises from an ongoing dialogue between the brain and the body.

We reinterpret the subconscious not as a repressed psychological domain, but as a valence-sensitive network of dynamic, embodied processes. It includes habits, affective responses, visceral memories, and autonomic regulation, sub-personal systems that operate beneath awareness but scaffold consciousness. These processes can be understood as the integration of somatic and tissue-level cognition into whole-body intelligence.

### Toward a unified framework: cognition across scales and material substrates

Bridging from sensing materials properties to cellular and whole-organism cognition enables a continuum view of mind, where intelligence emerges through material integration and organizational complexity. This perspective supports a non-dualistic account of mind, grounded in bio-physical systems and scaling up through recursive embodiment and feedback.

Importantly, this approach understands cognition as a property of organized responsiveness, not necessarily symbolic reasoning. It models the mind as a material phenomenon rooted in living matter, capable of being emulated in adaptive, embodied artificial systems.

### Implications for artificial general intelligence

Current AGI paradigms tend to replicate human-level symbolic reasoning, often abstracted from embodiment. A continuum theory of biological minds suggests that AGI should instead emulate the scalable, material, and embodied properties of natural cognition. From neuromorphic substrates to decentralized learning, lessons from biology may help construct systems with genuine adaptive intelligence.

Table 1 presents a spectrum of cognition, intelligence, awareness and mind forms across living systems, from bacteria to great apes and humans. It shows how cognitive capacities and behaviors increase in complexity with evolutionary development increasing organisms capacity to control their environment.

**Table 1:** A spectrum of cognition, intelligence, awareness and consciousness across life.

Organism	Cognition	Sentience	Intelligence	Awareness → consciousness	Mind
Bacteria	Distributed, chemical-based cognition (quorum sensing, gene regulation)	valenced behavior	Group-level intelligence (colony adaptation)	Minimal awareness, environmental sensing, no subjective experience	Self-regulating system
Fungi	Network-based, electrical signal processing, decentralized information integration	proto-sentience	Memory-like responses, decision-making in resource allocation	Environmental-awareness, responsive but not experiential, proto*- consciousness	Distributed mind
Plants	Chemical and electrical signaling, adaptive growth	Limited valenced responses	Environmental intelligence, adjusting structure based on stimuli	Limited awareness, sensitive to light, gravity, and touch but no subjective experience	Decentralized mind
Insects	Neural-based cognition, instinct-driven behavior	nociception and pain-like responses	Learning-based intelligence, simple problem-solving	Primary awareness, moment-to-moment experience, but no reflective thought	Functional limited mind
Cephalopods (octopuses, squids)	Complex, distributed neural cognition	strong sentience emotion-like states	High intelligence, tool use, learning, problem-solving	Likely conscious, flexible, possible self-awareness	Highly flexible mind
Birds and mammals	Advanced neural cognition, social learning, memory	Deep sentience (emotions, social bonds)	Social intelligence, problem-solving, tool use, reasoning	Conscious awareness, memory, emotional, self-recognition in some	Conscious mind
Great apes and humans	Abstract thought, symbolic reasoning, long-term planning	Deep sentience (emotions, social bonds)	Metacognition, cultural learning, language	Self-awareness, imagination, introspection, subjective experience	Fully conscious, reflective mind

*“proto” stands for early, first, from which other similar things develop.

By focusing on cognition as an emergent property of valence-sensitive organization—from molecules to organs to conscious experience, we find an alternative pathway for AGI development: one rooted in life’s own layered architecture of sense-making. Learning from nature, we focus on the self-organization and development of biological minds with increasing complexity of cognition, intelligence, sentience, awareness and consciousness.

## Definitions of cognition, intelligence, sentience, awareness, consciousness and mind in the unified framework

Creating definitions that apply to all life forms, we ensure they are broad enough to include both neural and non-neural organisms, precise enough to differentiate between levels of complexity and grounded in biological processes rather than making anthropocentric assumptions.

### On experiential terminology

Terms such as sentience, awareness, and experience are used in a graded and biologically grounded sense. At basal levels of life, these terms refer to functional, valence-sensitive regulation rather than to phenomenally conscious experience. Phenomenal consciousness, reflective awareness, and reportable subjective experience arise only under specific organizational conditions, typically involving nervous systems and integrative architectures. The use of experiential vocabulary at lower biological scales is not intended to attribute human-like subjective experience to single cells but to describe degrees of organized responsiveness relevant to the organism’s level of organization.

In what follows, the definitions of basic terms will be given and explained.

### Information

*Definition*: Information is a difference that makes a difference for the regulation of system–environment interactions.

Informational states are defined by their functional consequences for maintaining or modifying organized behavior, rather than by abstract symbols or semantic content. This allows information to be instantiated in biochemical, electrical, or mechanical configurations without presupposing representation or interpretation.

### Computation

*Definition*: Computation is the physical transformation of information through the intrinsic dynamics of a system (information dynamics).

On this view, computation need not involve symbolic manipulation or centralized control. It is realized by the material and dynamical properties of physical systems themselves (natural computation). Unlike conventional digital computation, which operates over abstract symbols in engineered architectures, morphological computation in living systems is realized through continuous physical dynamics of embodied structures. This interpretation is supported by work which demonstrates how embodied dynamics can perform computational functions without representation or explicit control architectures (e.g., Dodig-Crnkovic, 2012).

### Cognition

*Definition*: Cognition is the process by which an organism acquires, transforms, stores, and uses information to regulate its behavior and interactions with the environment.

Cognition exists in all life forms, from bacteria to humans. It includes information processing, sensory input, and response coordination (Dodig-Crnkovic, 2025). It does not require a brain. Bacteria, fungi, and plants exhibit cognition through chemical and electrical signaling. For example, bacteria use quorum sensing to make group decisions (Ng and Bassler, 2009; Waters and Bassler, 2005). Fungi transmit electrical signals across their mycelial networks (Adamatzky, 2022; Adamatzky et al., 2023; Baluška and Levin, 2016). Recent research on plant electrical and chemical signaling provides evidence for biological bases for plant sentience (Segundo-Ortin and Calvo, 2021). Animal cognition is studied in biosemiotics, ethology, comparative psychology, behavioral ecology, etc. and is well documented in mammals, birds, reptiles, fish and invertebrates, including insects (Greggers and Menzel, 1993).

### Sentience

*Definition*: Sentience is the capacity of an organism to have valenced responses—meaningful experiences of preference for beneficial conditions over harmful ones, that in the first step is valenced response that distinguishes “good” from “bad.” Sentience reflects a preference toward beneficial states. The organism does not just react but internalize inputs of individual/subjective experience. Sentience ranges from basic (bacteria avoiding toxins and move toward nutrients) (Lyon, 2015); insects exhibit pain-like responses to injury, (Gibbons et al., 2022) to complex (Ginsburg and Jablonka, 2019). Sentience does not require language or self-awareness.

Experiences are not neutral; they are perceived as “good” or “bad,” eliciting valenced responses. Sensory-based awareness implies that the organism processes sensory information in a way that affects behavior beyond pure reflexes. All living organisms possess a degree of sentience. Bacteria process information from the environment (cognition) and valuate it in terms of good/bad or attractive/repulsive. Table 2 presents types of sentience across life. All animals are sentient, but only some have self-awareness. Sentience is complex in organisms with nervous system, but simpler forms of sentience exist already in non-neural organisms.

**Table 2:** Types of “sentience” across life.

Life form	Type of valenced response	Type of sentience
Bacteria	Chemotaxis (moving toward nutrients, away from toxins)	Minimal sentience (goal-directed behavior)
Protists	Learning from negative stimuli, avoidance behavior	Sensory-based sentience (short-term memory, adaptive response)
Fungi	Memory-like growth preferences, adaptive decision-making	Decentralized valence-based awareness
Plants	Growth toward light (positive valence), toxin avoidance	Limited sensory awareness
Insects and simple animals	Active decision-making based on reinforcement	Basic sentience (nociception and pain-like responses)
Birds and mammals	Complex emotions, social intelligence	Higher-order sentience (affective experiences)

### Intelligence

*Definition*: Intelligence is the ability of an organism to learn, solve problems, and adapt behavior based on experience or environmental changes.

It is expressed on the individual level (octopus learning a task and using tools) (Godfrey-Smith, 2017) and collective level (bacteria in biofilms adapting to antibiotics) (Ben-Jacob, 2008; Ben-Jacob, 2009). Fungi adjust their growth patterns based on past and present nutrient availability (Fukasawa et al., 2024; Adamatzky et al., 2023). It does not require a brain—fungi and plants exhibit intelligence through adaptive behavior (Levin, 2023; Ginsburg and Jablonka, 2009). Bees learn and remember complex foraging routes (Greggers and Menzel, 1993).

### Awareness → consciousness continuum

*Definition*: Awareness → consciousness continuum is the ability of an organism to integrate sensory information, maintain a continuous state of responsiveness, and interact with the environment in a structured way.

This continuum ranges from basic environmental awareness to self-awareness and consciousness. It does not require thought or introspection—even bacteria and fungi are “aware” of their surroundings (Adamatzky et al., 2023). Simple organisms have sensory awareness, while complex organisms develop complex self-awareness and consciousness.

Bacteria detect chemical gradients and adjust behavior. Fungi sense nearby plants and redirect growth (Money, 2021). Dogs experience emotions and respond to social cues. Humans engage in self-reflection. In short, consciousness is not binary but a continuum, with different levels of complexity in different organisms (Damasio, 2010; Koch, 2012).

This graded perspective is related to what Ginsburg and Jablonka (2019) have described as the emergence of a “sensitive soul.” Within this view, the boundary between awareness and consciousness remains fluid and context-dependent. Some approaches place this boundary at the emergence of nervous systems, as they enable more integrated, flexible, and potentially self-referential forms of experience. In the present framework, awareness and consciousness are treated as points along a continuum of increasing organizational complexity.

## Biological evolution of consciousness as adaptation for life regulation

Damasio (2010) argues that consciousness is a process evolved as a biological strategy to enhance survival. At the heart of his process is homeostasis, the body’s ability to maintain internal balance. Simplest organisms began with non-conscious homeostatic regulation (e.g., basic responses to temperature, energy needs). Over time, feelings evolved as signals of the internal state, representing how well or poorly the body was doing (pleasure, pain, discomfort, etc.).

“Consciousness emerged from the development of increasingly complex and integrated representations of the organism’s state in relation to its environment and internal processes” (Damasio, 2010, pp. 99–105).

Damasio extends this argument in The Strange Order of Things (2018), showing that feelings associated with homeostatic regulation form the biological basis for value systems, including motivation, decision-making, and ultimately cultural practices.

Evolution selected for organisms that could feel how they were doing and adjust behavior accordingly. These feelings—rooted in homeostasis—became the “guiding light” of adaptive action. Over millions of years, these evolved into more sophisticated emotions, decision heuristics, and eventually social norms and cultural codes. “Feelings are the means by which nature found a way to make life regulation directly experienced by the organism” (Damasio, 2018, p. 8).

Damasio’s view is presented in Table 3, showing an evolutionary ladder from proto-self to extended consciousness.

**Table 3:** An evolutionary ladder of consciousness according to Damasio.

Level	Description	Evolutionary significance
Proto-self	Non-conscious representation of the body	Present in simple organisms
Core consciousness	Awareness of the present moment; feeling of existence	Present in many animals
Extended consciousness	Autobiographical self, memory, identity over time	More recent in primates and humans

The table reflects the fact that “The biological roots of self and consciousness are found in the same mechanisms that underlie life regulation” (Damasio, 2010, p. 23).

### Mind

Just like cognition, sentience, intelligence, and awareness → consciousness, the term “mind” is currently ill-defined and anthropocentric. If we want definitions that apply to all living organisms, and generalize to machines, we need generalized but precise explanations that allow for different levels of complexity across species.

*Definition*: Mind is the activity of an organism that processes information, integrates sensory input, regulates internal states, and generates adaptive responses.

The mind is the totality of cognitive, sentient, intelligent, and conscious functions working together.

This definition points out the dynamical and multi-tasking aspects of mind, which includes information processing (like cognition), regulation of behavior and adaptation (like intelligence) and integration of internal and external signals (like awareness).

The key features of a mind are information processing, signal integration, behavior regulation and adaptation (Maturana and Varela, 1980). Mind is not present only in a physical brain—it includes all information-processing mechanisms. It applies to both centralized (brains) and distributed/decentralized (fungal networks) systems. It exists in all living systems as a means they regulate themselves.

For example, fungal networks process information about nutrients and threats (Adamatzky et al., 2023; Money, 2021). Insect colonies function as “collective minds” that solve problems (Almér et al., 2015). Human brains engage in complex reasoning and creativity.

All biological organisms consist of cells, so “cellular minds” are the building blocks for all living minds, including human. Living organisms at different levels of complexity possess different forms of cognition and intelligence adapted to their environment. Awareness exists in many forms, from simple environmental sensing to deep introspection.

Brains are not required for cognition. Cognition is distributed information processing. Brains as well process distributed information. Consciousness appears on a continuum, with different levels of experience in different organisms.

### The unified model of biological minds

Looking at processes of cognition, sentience, intelligence, awareness → consciousness and mind as a process integrating them all (Table 4) we see how those properties constitute a unified view of mind in living systems.

**Table 4:** Unified model of mind with its different aspects and functions.

Concept	Description	Applies to	Key function
Cognition	Acquiring, processing, and using information	All living organisms	Forms the foundational regulatory layer enabling organism–environment interaction
Sentience	The ability to have valenced responses (seeking beneficial conditions, avoiding harm)	Many organisms, from bacteria to mammals	Assigns value (good/bad) to states, guiding preference, avoidance, and motivation
Intelligence	Learning, problem-solving, and adaptation	Organisms that adjust behavior based on experience	Enhances decision-making through learning and flexible adjustment over time
Awareness → consciousness	Integration of sensory information for structured responsiveness	All living organisms, in graded forms	Integrates experience into coherent responsiveness; in complex organisms, supports subjective experience
Mind	The organized activity integrating cognition, sentience, intelligence, and awareness	All organisms with organized behavior	Constitutes the dynamic, unified process enabling perception, regulation, and adaptive action

### A diversity of minds

Instead of asking “Which organisms are conscious?” or “Which organisms are intelligent?,” the “diverse minds” approach suggests a more nuanced view. Different organisms exhibit different forms of cognition, adapted to their ecological needs. Intelligence emerges in multiple ways, through centralized brains or decentralized networks. Consciousness is a spectrum, without a strict boundary, with varying degrees of “subjective” (individual) experience. This understanding has a wide range of consequences.

### Different life forms in the unified mind model

Even simple life forms have a “mind” if we define it as the system integrating level of cognition, intelligence, and awareness. Evolution leads to higher minds developed with greater intelligence and more complex consciousness (Jablonka et al., 2014).

Instead of being a physical object, the mind is an emergent process—it arises when cognition, sentience, intelligence, and consciousness interact in a dynamic, self-regulating way. This means that minds can exist in decentralized systems such as bacteria colonies and fungi (Lyon, 2015; Adamatzky, 2022). Minds can exist without self-awareness as in plants and insects. The complexity of a mind depends on the depth of cognition, intelligence, and consciousness.

As biological minds exist on a spectrum, we can categorize organisms by the depth of their mind-related capacities. Instead of asking “Which organisms have minds?,” a better question is: “What kind of mind does an organism have?” The mind is not an object but a process— an active, embodied, predictive interface between organism and world (Clark, 2016). It exists in different degrees across all life forms.

## The info-computational model for the mind in the unified framework: relating cognition, sentience, intelligence, and awareness → consciousness

The proposed continuum of cognition is supported by two complementary theoretical paradigms that converge on the naturalization of mind and intelligence: the Info-Computational framework (Dodig-Crnkovic, 2012, 2016, 2018, 2025) and the Free Energy Principle (Friston, 2010, 2013). Both approaches reject Cartesian dualist view of mind vs. matter and emphasize that cognitive processes are grounded in the same organizational principles that sustain life.

In the Info-Computational framework, living systems are understood as morphological information processors (Dodig-Crnkovic, 2012, 2018), their physical organization embodies computation. Morphology, from molecular to macroscopic scales, determines how matter encodes, transforms, and acts upon information. Computation is thus not only symbolic or neural but constitutive of life itself, including its simplest forms (Dodig-Crnkovic, 2025). Each organism processes information thus computes its own survival by integrating environmental inputs, internal states, and past experiences into adaptive change. In this sense, biology is computation in action, and cognition arises from the dynamic coupling between information structures and their physical realization. As an example, Ehresmann (2012) provides a mathematical info-computational model for (neuro-) cognitive systems capable of creativity.

The Free Energy Principle with active inference describes how biological systems preserve their organization through variational self-regulation (Friston, 2010, 2013). Organisms actively minimize a quantity known as free energy, which measures the divergence between predicted and sensed states. This process provides a unified account of perception, learning, and action, in a form of predictive inference through which living systems maintain their dynamical integrity within an uncertain world.

These two complementary paradigms describe a morphogenetic-predictive loop that spans from basal cognitive systems in non-neural organisms to advanced reflective awareness in humans. Morphological computation provides the material substrate, while predictive dynamics establish the inferential processes that turn structure into process of cognition. The continuum of mind, therefore, can be seen as a nested hierarchy of information-processing systems that maintain their viability through predictive embodiment.

Info-Computational naturalism and the Free Energy Principle offer a coherent theoretical foundation for de-anthropomorphizing cognition. They characterize intelligence and cognition not as peculiarity of complex brains but as natural outcomes of life’s universal imperative: to model, predict, and regulate one’s own existence within an ever-changing environment.

## Evolution, natural selection, the extended evolutionary synthesis, and natural induction

The continuum of cognition and adaptive organization proposed here aligns with recent expansions of evolutionary theory under the Extended Evolutionary Synthesis (EES) and with the emerging concept of natural induction (Buckley et al., 2024). Classical Neo-Darwinian frameworks have long regarded natural selection as the sole mechanism capable of generating spontaneous adaptive organization. However, both the EES and the natural induction paradigm reveal that adaptive complexity can also emerge from intrinsic physical and developmental processes that predate, complement, and even operate independently of selection.

The EES extends Darwin’s original account by recognizing the generative roles of developmental bias, phenotypic plasticity, niche construction, and extended inheritance (Laland et al., 2015; Levin, 2023). These processes incorporate self-organization and agency in biological systems as natural sources of adaptive form. Rather than treating selection as the sole mechanism of design, the EES reframes evolution as a multilevel, reciprocal interaction between organisms and their environments, mediated by complex feedback among development, physiology, and ecological dynamics. This view resonates with the info-computational framework, in which morphology, environment, and information flow co-constitute the evolving system’s capacity for sense-making.

Buckley et al. (2024) propose natural induction as a complementary form of spontaneous adaptive organization that can occur even in the absence of reproduction or selection. Natural induction arises from dynamical feedback between physical optimization and learning in systems described by networks of viscoelastic connections. When such networks experience intermittent disturbance, their structure gradually adapts to reduce internal frustration, giving rise to non-trivial adaptation—systems that literally learn how to optimize better with experience. This process provides a purely physical mechanism for adaptation through the differential easing of internal constraints, rather than through differential survival and replication.

In this light, the EES and natural induction jointly articulate a broadened view of evolution and adaptation as emergent phenomena of embodied, informational, and iterative systems. Natural selection remains a central macroscopic process of population-level organization, while natural induction models adaptive tendencies that can also originate below the level of reproduction, in the *morphogenetic and material dynamics that allow matter to learn and reorganize*. The interaction of these processes offers a richer evolutionary continuum—from morphological computation and predictive free-energy minimization at the physical-embodied level (Friston, 2010, 2013; Dodig-Crnkovic, 2018; Dodig-Crnkovic, 2025), through developmental bias and plasticity at the organismic level, to Darwinian selection at the ecological and population level.

Denis Noble’s theory of biological relativity (Noble, 2012) challenges gene-centric evolutionary views by arguing that causation in biology is multi-level and reciprocal rather than unidirectional. Higher biological levels exert top-down influences on lower levels like genes, constituting circular causation without any privileged causal locus. This framework expands the understanding of evolutionary processes to incorporate dynamic systemic interactions. Noble’s ideas align well with his recent collaborative work on natural induction (Buckley et al., 2024), advancing an evolutionary paradigm where spontaneous adaptive organization emerges through physical learning and network dynamics beyond classical natural selection.

Together, Noble’s relativity and Watson’s natural induction frame evolution as a live, systemic process of ongoing learning and organization—one that transitions seamlessly from molecular, cellular, tissues, organs, to organismal levels, driven by physical and informational principles rather than solely by random mutations and differential reproduction.

Integrating these perspectives situates biological cognition and intelligence within a unified hierarchy of natural information processing, in which adaptive agency, at multiple scales, emerges from recursive interactions of physical learning, predictive modeling, and environmental coupling. Under this view, life evolves not only by selection of the fittest, but also by self-organizing induction of the fitted, *through iterative*, *embodied computation that continuously refines the correspondence between internal dynamic models and external constraints* (Table 5).

**Table 5:** Unified cognition across levels of biological organization (ICON framework).

Level of biological organization	Cognitive characteristics (ICON interpretation)
Single cells (e.g., bacteria, unicellular eukaryotes)	Behaviorally expressed regulation; valence-sensitive states; learning as history-dependent physiological change; minimal organism-relative experience
Multicellular tissues and simple multicellular organisms (e.g., biofilms, plants, simple metazoa)	Distributed coordination; collective learning and memory; adaptive morphogenesis; tissue-level experience expressed as pattern stability and error correction
Organisms with simple nervous systems (e.g., insects, worms)	Integrated behavior; learning and memory; sleep and internal state regulation; nociception and pain-like responses; structured organism-level experience
Organisms with complex nervous systems (e.g., vertebrates, mammals)	Flexible learning; affective states; extended experience; awareness; goal-directed behavior across longer time horizons
Humans	Symbolic cognition; language; reflective awareness; abstract reasoning; culturally and socially scaffolded experience

### Related frameworks

Empirically motivated proposals for unified frameworks of cognition across living systems have emerged from observations of cognitive-like behavior spanning biological scales (Dennett, 2017; Levin, 2022, 2023). However, these proposals have not articulated a systematic explanatory account of the generative mechanisms through which diverse cognitive capacities arise and relate to one another within a single coherent framework.

A related attempt at unifying mind and matter is Nakajima’s cognizers system (CS) model, which extends the notion of cognition hierarchically across physical, chemical, and semiotic levels by defining cognition as a relational state change (“related state change”) (Nakajima, 2024). Within that framework, semiotic cognition is emphasized as the level most closely aligned with conventional cognitive and mental phenomena, while physical and chemical “cognitions” describe context-dependent state transitions in nonliving systems. The present work (ICON) shares the aim of theoretical unification, but differs in locating cognition primarily at the level of living organization and treating behavior, learning, and minimal experience as present from the onset of life and progressively complexified with increasing biological organization. ICON locates cognition at the level of biological organization, grounding meaning and normativity directly in the viability conditions of living systems. Physical and chemical substrates support cognitive functions such as memory, recall, information processing, and communication. Table 6 presents the comparison between ICON and CS models.

**Table 6:** ICON framework vs. CS model of Nakajima.

Aspect	ICON (Dodig-Crnkovic)	CS model (Nakajima)
Starting point	Life	World
Cognition defined as	Viability-oriented behavior, learning, experience	Related state change
Cognition present in	All living systems	Physical, chemical, and semiotic entities
Experience	Present from the start (minimally)	Emerges at semiotic level
Awareness	Higher-order organization	product of internal symbolic processes that infer external conditions
Organization	Continuous elaboration	Hierarchical extension
Meaning	Intrinsic to life	Semiotic and symbolic

### Empirical consequences and testability

Identifying cognition with living organization establishes a two-fold empirical connection: *an explanatory role*, in which diverse biological phenomena are unified under a common organizational account, and *a predictive role*, in which the framework constrains expectations about how adaptive behavior should change under experimental conditions.

Explanatorily, the framework accounts for empirical findings that are difficult to reconcile with current views restricting cognition to neural or representational architectures. In particular, work on bioelectric regulation of morphogenesis demonstrates that multicellular tissues can store, recall, and correct target anatomical patterns through distributed physiological networks, independently of nervous systems (Levin, 2014; Whited and Levin, 2019). Such phenomena exhibit error correction, goal-directed regulation, and context sensitivity, and are therefore more naturally understood as manifestations of multiscale organizational cognition than as the by-products of local molecular dynamics alone.

Predictively, the framework yields empirically testable expectations. If cognition depends on self-maintaining, multiscale organization rather than on localized information processing alone, then systematic disruption of organizational coupling, such as perturbations of gene regulatory networks, bioelectric signaling, or intercellular connectivity, should impair adaptive regulation even when local signaling mechanisms remain intact. This logic is consistent with experimental results showing loss of morphogenetic competence and stable anatomical outcomes following targeted disruption of bioelectric patterning and physiological coordination (Levin, 2014; Whited and Levin, 2019), and with predictive-processing interpretations of organisms as problem-solving systems rather than passive stimulus–response machines (Pezzulo and Levin, 2016).

Established paradigms in developmental and regenerative biology provide concrete test cases for these predictions. The remarkable regenerative abilities of planarian flatworms, such as whole-body regeneration from small tissue fragments, cannot be explained solely by local gene expression or molecular gradients. Experimental work shows that these competences depend on long-range bioelectric signaling and forms of anatomical memory that enable error-correcting pattern regulation (Levin, 2014; Whited and Levin, 2019) which exact mechanisms remain underexplored. Within the proposed framework, such phenomena are predicted to arise from cognitive capacities of cell collectives, whereby cells store, evaluate, and update information about target morphology and adjust their behavior accordingly, which reframes theoretical understanding in a cognitive–organizational terms (Levin, 2022).

In this way, the proposed framework links explanatory unification with experimentally grounded predictions, situating cognition as a property of living organization that is already under active empirical investigation.

## Conclusion

The exploration of cognition, intelligence, sentience, and consciousness across biological systems shows that mind is a continuum of cognitive processes, adapted to ecological and evolutionary contexts across successive levels of organization. By moving beyond anthropocentric definitions, we can recognize diverse forms of intelligence and awareness in organisms ranging from bacteria to mammals, each exhibiting unique mechanisms of information processing, including decision-making, and adaptive behavior.

This perspective challenges traditional assumptions about mind and what it means to be “intelligent” or “conscious” and has profound implications for multiple disciplines. In biology, it encourages a more nuanced understanding of cognition across species. In artificial intelligence, it inspires new approaches to machine learning and autonomous systems by recognizing intelligence beyond centralized neural structures. In philosophy and ethics, it invites reconsideration of concerns for non-human life forms based on their capacity for experience and cognition.

Future research should focus on empirically testing the proposed unified model of cognition and intelligence in both biological and artificial systems. By refining our definitions and methodologies, we can develop a more comprehensive framework that not only enhances our understanding of natural intelligence but also informs the design of future intelligent technologies.

Ultimately, de-anthropomorphizing mind allows for a richer, more inclusive approach to studying cognition—one that respects the complexity and diversity of minds across the natural world and the broader space of possible minds.

## Data Availability

The original contributions presented in the study are included in the article/supplementary material, further inquiries can be directed to the corresponding author.
